# FDG-PET/CT imaging for staging and radiotherapy treatment planning of head and neck carcinoma

**DOI:** 10.1186/1748-717X-3-29

**Published:** 2008-09-18

**Authors:** Letizia Deantonio, Debora Beldì, Giuseppina Gambaro, Gianfranco Loi, Marco Brambilla, Eugenio Inglese, Marco Krengli

**Affiliations:** 1 Radiotherapy, University of Piemonte Orientale "Amedeo Avogadro", Novara, Italy; 2 Medical Physics, Hospital Maggiore della Carità, Novara, Italy; 3 Nuclear Medicine, University of Piemonte Orientale "Amedeo Avogadro", Novara, Italy

## Abstract

**Background:**

Positron emission tomography (PET) has a potential improvement for staging and radiation treatment planning of various tumor sites. We analyzed the use of ^18^F-fluorodeoxyglucose (FDG)-PET/computed tomography (CT) images for staging and target volume delineation of patients with head and neck carcinoma candidates for radiotherapy.

**Methods:**

Twenty-two patients candidates for primary radiotherapy, who did not receive any curative surgery, underwent both CT and PET/CT simulation. Gross Tumor Volume (GTV) was contoured on CT (CT-GTV), PET (PET-GTV), and PET/CT images (PET/CT-GTV). The resulting volumes were analyzed and compared.

**Results:**

Based on PET/CT, changes in TNM categories and clinical stage occurred in 5/22 cases (22%). The difference between CT-GTV and PET-GTV was not statistically significant (p = 0.2) whereas the difference between the composite volume (PET/CT-GTV) and CT-GTV was statistically significant (p < 0.0001).

**Conclusion:**

PET/CT fusion images could have a potential impact on both tumor staging and treatment planning.

## Background

In squamous cell carcinoma of the head and neck, definitive radiotherapy (RT) provides improved disease control and survival rates through high dose radiation and concurrent administration of systemic drugs. The recent introduction of sophisticated technology, like intensity modulated radiotherapy (IMRT), promises to improve the cost/benefit ratio of therapy further [1,2].

The precise identification of tumor volume remains an open issue: tumor dose escalation and sparing of normal tissue requires a precise identification of the extension of the disease in individual patients. For these reasons, radiation oncologists are interested investigating functional imaging such as positron emission tomography (PET) in particular with ^18^F-fluorodeoxyglucose (FDG) that provides improved staging, treatment response identification, and recurrence detection for a wide range of solid cancers [3,4], including head and neck carcinoma [5,6]. FDG-PET, however, requires careful correlation with structural images for precise tumor localization because of lack of anatomical information. FDG-PET images can be directly incorporated into CT-based RT planning through a variety of image registration strategies [7,8]. This allows radiation oncologists to use the complementary strengths of functional (PET) and structural imaging (CT) co-registered in a single image set.

The purpose of this study was to investigate the potential impact of using PET/CT image fusion for the management of patients with head and neck carcinoma. Specifically, we analyzed how PET/CT may change the clinical stage and the delineation of gross tumor volume (GTV) for radiation treatment planning.

## Methods

### Patients characteristics

Twenty-two consecutive patients with primary head and neck carcinoma were selected for radiotherapy after discussion in multidisciplinary conference and obtaining informed consent following the rules of our institution. Patients with Karnofsky performance status < 80/100, tumor location in the salivary glands or unknown primary site, evidence of distant metastases at initial staging, and need for surgical procedures were excluded from this study. Clinical characteristics of these patients are summarized in Table 1. All patients underwent routine workup including clinical examination, fiber-endoscopy, contrast-enhanced CT of the head and neck district, chest X-rays, and liver ultrasound (US). The clinical stage was defined according to the 2002 American Joint Committee on Cancer-International Union Against Cancer (AJCC-UICC) classification [9]. No patients were candidates for curative surgery. Fifteen patients were candidates for combined radiotherapy and platinum-based chemotherapy and 7 patients for radiotherapy alone.

**Table 1:** Characteristics of study population.

Characteristics	N.
*Patients*	22
*Age (years)*	
Median	59
Range	43–76
*Gender*	
Male	18
Female	4
*Tumor subsite*	
Oral Cavity	2
Oropharynx	6
Hypopharynx	6
Larynx	2
Nasopharynx	4
Paranasal sinuses	2
*Histology*	
Squamous cell	18
Undifferentiated	3
Adenocarcinoma	1
*Pathologic AJCC stage*	
I	4
II	5
IIB	2
III	3
IVA	7
IVB	1
*Pathologic TNM categories*	
T1, T2, T2b, T3, T4a N0 M0	13
T2, T4a N1 M0	2
T2 N2a M0	1
T2, T3, T4a N2b M0	5
T4 N3 M0	1

Abbreviations: AJCC: American Joint Committee on Cancer; T: tumor extension; N: lymph-nodal disease; M: distant metastasis.

### Image acquisition and fusion

All patients underwent routine CT simulation in supine position, immobilized with head-rest and customized thermoplastic mask by using the scanner GE Prospeed (General Electric, Milwaukee, WI, USA). The planning volume was scanned from the top of the skull to the mid-thorax. The CT simulation images were subsequently fused to the hybrid PET/CT images by means of a dedicated radiation treatment planning system (RTPS) image fusion tool (Syntegra, Philips Medical System, Eindhoven, The Netherlands) based on a mutual information algorithm.

PET/CT was performed within 5 working days from the CT simulation scan and after at least 21 days from the tumor biopsy. In order to assure a reproducible patient setup, the same immobilization device used during CT simulation as well as a flat-panel carbon fiber composite table insert were also used for PET/CT acquisition.

Images were acquired by the Biograph 16 HI-REZ PET/CT scanner (Siemens, Hoffman Estates, IL, USA). The PET component is a high-resolution scanner with a spatial resolution of 4.7 mm and no septa, thus allowing 3D-only acquisitions. The CT component is the Somatom Sensation sixteen-slice CT (Siemens, Hoffman Estates, IL, USA). The CT scanner is used both for attenuation correction of PET data and for localization of FDG uptake in PET images. All patients were advised to fast for at least 6 hours prior to PET/CT examination. After injection of about 5 MBq of FDG per kg of body weight, patients were rested for a period of about 60 minutes in a comfortable chair. Emission images ranging from the proximal femur to the base of the skull were acquired for 2–3 minutes per bed position. Field of view was of 50 cm with a matrix of 512 × 512 pixels for CT and of 128 × 128 for PET. The processed images were displayed in coronal, transverse, and sagittal plans. After image acquisition, PET/CT data sets were sent to the treatment planning system Pinnacle (Philips, Adac Laboratories, Milpitas, CA, USA) through local network.

### Tumor staging and target volume delineation

The clinical staging was analyzed by comparing the PET/CT with the CT alone findings. For PET image interpretation, a focal FDG uptake was considered as positive when the activity was significantly above the expected background and could not be explained by a normal structure. A fixed image intensity threshold method (40% of maximum intensity) was used to outline the PET-GTV for the primary tumor and the involved nodal sites [10].

The target volumes were outlined by two radiation oncologists with specific experience in head and neck tumors management according to the guidelines of the International Commission on Radiation Units and Measurements Report 62 [11]. They were not blinded to each other and outlined together the contours achieving a final consensus. GTVs were contoured on CT images obtaining CT-GTV, PET (PET-GTV), and PET/CT images (PET/CT-GTV). The PET/CT-GTV included both PET and CT information. The clinical target volume (CTV), for treatment purpose, was identified as the PET/CT-GTV with an additional 5 mm margin and included also the regional lymph nodes. The planning target volume (PTV) was subsequently semi-automatically outlined giving an additional 5 mm margin to the CTV.

For clinical purposes, we always considered the PET image as an additional information to CT image either for tumor staging or for target contouring for treatment planning.

### Statistical analysis

The difference between PET-GTV and CT-GTV and between PET/CT-GTV and CT-GTV were analyzed by Wilcoxon signed rank test. A *p *value of less than 0.05 was considered to be statistically significant. Data were reported as mean ± 95% confidence interval.

**Figure 1:**
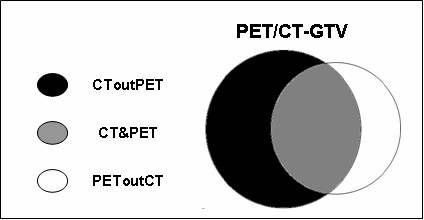
**The diagram shows the volumes identified after fusion of CT and PET images**. "PET/CT-GTV" is the composite volume of PET and CT; "PEToutCT" is the volume identified by PET but not by CT; "CToutPET" is the volume identified by CT but not by PET; "CT&PET" is the common volume of PET and CT.

The following additional volumes were considered for the statistical analysis (Figure 1):

-the volume identified by PET but not by CT (PEToutCT),

-the volume identified by CT but not by PET (CToutPET),

-the common volume of CT and PET (CT&PET).

## Results

### Tumor staging

PET/CT imaging lead to a change in the TNM categories and in the clinical stage in 5/22 (22%) cases compared to CT alone (Table 2). T-stage changed in 3 of 22 (14%) and N-stage in 2 of 22 cases (10%) N0 at CT. In one of these cases (case 3 in Table 2), the PET/CT finding was confirmed by fine needle agobiopsy (FNA). This patient was affected by squamous cell carcinoma of the left hypopharyngeal wall with 1 cm in diameter lymph node in the left level II had intense FDG uptake suggestive for nodal involvement with potential upstaging from N0 to N1. In such a case, biopsy did not find the presence of tumor cells and the patient was considered as N0. In another case of hypopharyngeal cancer (case 5 in Table 2), a lymph node in the mediastinum, suggestive for metastatic disease, although not confirmed with biopsy by mediastinoscopy because of medical contraindications for general anesthesia, was detected by PET/CT. In this case, the clinical stage changed from M0 to M1 and consequently the treatment intent from curative to palliative. This patient received radiotherapy combined with chemotherapy and died with intra-thoracic and liver metastases 8 months later.

**Table 2:** Change of clinical stage related to PET/CT in 5/22 patients (22%).

Case N.	Site	CT stage	PET/CT stage
1	Oropharynx	T1 N0 M0	**T2 N0 M0**
		Stage I	**Stage II**
2	Nasopharynx	T3 N2b M0	**T4 N2b M0**
		Stage III	**Stage IVA**
3	Hypopharynx	T1 N0 M0	**T1 N1 M0**
		Stage I	**Stage III**
4	Hypopharynx	T2 N0 M0	**T2 N1 M0**
		Stage II	**Stage III**
5	Hypopharynx	T3 N2b M0	**T4 N2b M1**
		Stage IVA	**Stage IVC**

*Abbreviations*:

CT: computed -tomography;

PET/CT: positron emission tomography

### Target volumes

**Figure 2:**
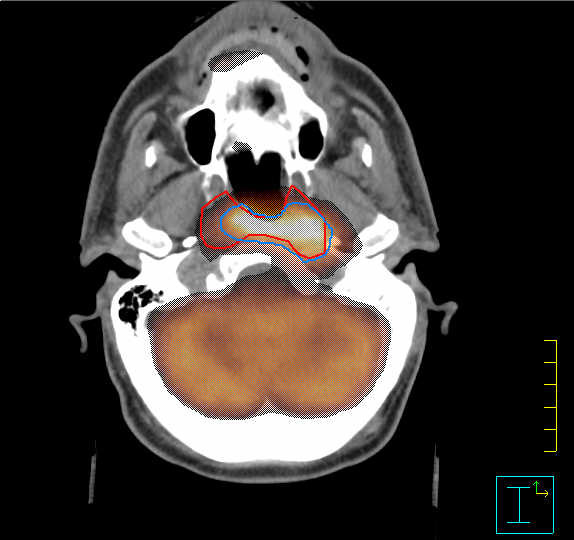
**Axial FDG-PET/CT image of a patient with nasopharyngeal undifferentiated carcinoma**. The computed tomography gross tumor volume (CT-GTV) and the positron emission tomography (PET)-GTV are highlighted with red and light blue contours, respectively. For treatment purposes both findings were taken into account.

As for the volume delineation, PET-GTV was smaller than CT-GTV (17.2 cc, with a standard deviation of 16.8 cc vs. 20.0 cc, with a standard deviation of 17.8 cc) with a mean difference of 2.8 cc, that was not statistically significant (p = 0.2). However, PET/CT-GTV (26 cc), that was used for clinical purposes, was significantly greater than CT-GTV (p < 0.0001). These volumes had a mean difference of 6 cc (Figure 2). The analyzed volumes for all patients are reported in Table 3.

**Table 3:** Volumes (cc) identified by CT and PET in every single case.

Patients	Subsites	CT-GTV	PET-GTV	PET/CT-GTV
1	Nasopharynx	17.7	30.3	36.5
2	Oral Cavity	65.9	49.0	78.7
3	Oral Cavity	16.1	22.2	25.1
4	Oropharyx	52.5	34.1	60.2
5	Hypopharynx	5.4	1.7	5.4
6	Hypopharynx	45.5	46.3	64.2
7	Hypopharynx	8.7	12.1	13.5
8	Oropharynx	35.3	20.1	36.1
9	Nasopharynx	6.3	3.5	6.4
10	Paranasal sinus	17.4	8.0	17.8
11	Oropharynx	2.1	4.5	5.3
12	Larynx	9.8	1.7	9.8
13	Nasopharynx	37.0	57.8	60.3
14	Oropharynx	6.0	2.6	6.2
15	Oropharynx	28.9	11.2	29.4
16	Oropharynx	6.5	17.3	19.8
17	Hypopharynx	13.4	4.7	13.9
18	Hypopharynx	1.3	4.0	5.0
19	Nasopharynx	17.8	15.2	20.3
20	Paranasal Sinus	40.8	34.7	61.8
21	Hypopharynx	7.1	8.9	11.9
22	Larynx	19.5	6.5	19.5

The mean and range values of the additional volumes analyzed to compare PET/CT and CT alone are reported in Table 4 and Figure 1. In particular, the mean PEToutCT volume was 27% of the mean CT-GTV and resulted ≥ 10%, i.e. 2 cc, larger than the mean CT-GTV in 13/22 patients (59%).

**Table 4:** Volumes (cc) identified after fusion of PET and CT images.

Volumes	Mean	Range	Confidence Interval
PET/CT-GTV	26.0	5.0–78.7	15.9–36.2
PEToutCT	5.5	0.0–21.2	2.4–8.7
CToutPET	8.1	0.7–28.0	4.4–11.8
CT&PET	11.2	0.4–36.2	6.2–16.2

Abbreviations: PET/CT-GTV: the composite volume between PET and CT; PEToutCT: the volume identified by PET but not by CT; CToutPET: the volume identified by CT but not by PET; CT&PET: the common volume of the two image modalities (CT and PET).

## Discussion

The use of FDG-PET/CT prior to treatment has gained interest in the radiation oncology community in relation to a potential improvement of tumor staging, optimization of treatment strategy, and better delineation of target volume[12].

The advantage of PET/CT fusion has been already reported for staging and RT planning of non-small cell lung cancer in a number of literature studies [13] and preliminary data on the role of PET/CT fusion are available for other tumor locations such as esophagus, rectum, anal canal, and pancreas [14-16].

Recent results in a limited number of studies are available about the role of PET/CT imaging for staging and RT treatment planning also of patients with head and neck carcinoma [17-20].

In our experience, the primary tumor was identified by PET/CT in all 22 patients with a change for both TNM categories and clinical stage in 22% of them. Similar results were reported by Koshy and Paulino [21]. In their study, they enrolled 36 patients and found that PET/CT fusion altered the TNM categories in 38% and the clinical stage in 14% of the patients. Also in a recent study published by Wang et al. [22], the CT-based staging was changed by PET/CT in 16/28 cases (57%).

In the second part of our study, we evaluated how PET/CT may influence the delineation of GTV considering the PET information as additional to that of CT. In our experience, the tumor volume identified by PET only was smaller than CT-GTV but the co-registration of PET and CT images allowed the identification of a potentially greater GTV, used for clinical purposes, similarly to what observed by Schwartz et al. [2] in 19 patients. In our series, the PET-GTV was smaller than the CT-GTV but the difference (2.8 cc) was not statistically significant (p = 0.2), whereas Heron et al. [18] found a significant decrease (p = 0.002) of PET-based GTV compared to CT-based GTV with a median difference of 22.3 cc in a group of 21 patients. Similarly, Paulino et al. [23] observed that PET-GTV was smaller in 75% of 40 patients, with a median difference between CT-GTV and PET-GTV of 16.9 cc. The finding of CT-GTV larger than PET-GTV in our as well as in other published series may be related to areas of necrosis inside the tumor identified by CT but not by PET because of lack of FDG uptake of the necrotic tissue.

A possible criticism to most of these studies, including the present one, is the uncertain correlation of the PET/CT findings with the real tumor extension that can only be precisely assessed on the surgical specimen. In our study, only 1 case had a cytological correlation: PET/CT overestimated the lymph nodal tumor extension. The correlation between tumor delineation on PET compared to pathology was investigated by Halpern et al. [24] who compared FDG-PET/CT image fusion with histopathology on 49 patients. A patient-by-patient analysis yielded a sensibility of 88%, a specificity of 78%, and an accuracy of 86% for PET/CT compared to pathology. In another study, Daisne et al. [25] compared the GTV identified by PET/CT with the pathology specimen of 9 patients affected by head and neck carcinoma. The investigators observed that the GTV delineated on the pathology specimen was in average smaller than that identified by PET/CT. In particular, PET/CT underestimated part of the macroscopic tumor extension in the mucosa of the contralateral larynx but, on the other hand, overestimated the infiltration of the cartilage, the extra laryngeal and pre-epiglottic space, and the thyroid gland.

In the article of Daisne et al. [25], the average GTV identified only by PET corresponded to 14% of the GTV contoured from CT images for oropharyngeal and to 13% for laryngeal and hypopharyngeal tumor locations. In our study, such value was as high as 27%. This fact should be carefully taken into consideration because the inclusion of this mismatched volume in the target volume could reduce the incidence of geographical missing. This additional volume identified only by PET may have an even greater importance when using highly conformal techniques like intensity modulated radiation therapy (IMRT), stereotactic radiotherapy, and charged particle therapy.

A relevant still open issue is the consistency of target delineation on PET images. In the available literature, GTV contouring with FDG-PET has varied, but has typically been based on standard uptake value (SUV). In the present study, we adopted the 40% of the SUV similarly to what proposed by other authors for lung and also head and neck tumors [10,26,27].

## Conclusion

The present study shows that FDG-PET/CT images for primary head and neck carcinoma had a potential impact on both tumor staging and treatment planning. A clinical stage variation was observed in 22% of cases and a significant greater GTV was detected thanks to PET/CT images. Based on our data as well as the other literature results, the future scenario of imaging for radiotherapy of head and neck tumors may include the use of functional imaging such as FDG-PET/CT with the aim to characterize the biological features of the tumor and optimize the use of highly conformal and biologically effective radiation treatment.

## Competing interests

The authors declare that they have no competing interests.

## Authors' contributions

LD is the study coordinator, participated in the development of the study and drafted the manuscript. GL worked on analysis of data, DB, GG, EI and MB participated in the design of the study and are involved in continuing optimization. MK is the study chairman, developed the design of the study, is involved in continuing optimization and helped to draft the manuscript. All authors read and approved the final manuscript.
